# Supplementary material for: Developmental Roles of FUSE Binding Protein 1 (Fubp1) in Tooth Morphogenesis
Source: Int J Mol Sci. 2020 Oct 29;21(21):8079. doi: 10.3390/ijms21218079 (PMC7663687; doi:10.3390/ijms21218079)
Supplement: Supplementary file 1 [file ijms-21-08079-s001.pdf]

Developmental roles of FUSE Binding Protein 1 (*Fubp1*) in tooth morphogenesis

Yam Prasad Aryal[1], Sanjiv Neupane[1], Tae-Young Kim[1], Eui-Seon Lee[1], Nitin Kumar Pokhrel[1], Chang-Yeol Yeon[1], Ji-Youn Kim[2], Chang-Hyeon An[3], Seo-Young An[3], Eui-Kyun Park[4], Jung-Hong Ha[5], Jae-Kwang Jung[6], Hitoshi Yamamoto[7], Sung-Won Cho[8], Sanggyu Lee[9], Do-Yeon Kim[10], Tae-Yub Kwon[11], Youngkyun Lee[1], Wern-Joo Sohn[12], Jae-Young Kim*[1]

[1]Department of Biochemistry, [3]Department of Oral and Maxillofacial Radiology, [4]Department of Oral Pathology and Regenerative Medicine, [5]Department of Conservative Dentistry, [6]Department of Oral Medicine, [10]Department of Pharmacology, [11]Department of Dental Biomaterials, School of Dentistry, IHBR, Kyungpook National University, 2177, Dalgubeol-daero, Jung-gu, Daegu, 41940, Korea

[2]Department of Dental Hygiene, Gachon University, Incheon, Korea

[7]Department of Histology and Developmental Biology, Tokyo Dental College, Tokyo, Japan

[8]Division in Anatomy and Developmental Biology, Department of Oral Biology, Yonsei University College of Dentistry, Seoul, Korea

[9]School of Life Science and Biotechnology, Kyungpook National University, Daegu, Korea

[12]Pre-Major of Cosmetics and Pharmaceutics, Daegu Haany University, Gyeongsan, Korea

*Corresponding address:

Jae-Young Kim, 2177 Dalgubeol-daero, Joon-gu, Daegu 41940, Korea

Tel: +82-53-420-4998; E-mail: jykim91@knu.ac.kr

ORCID: 0000-0002-6752-5683

n=8	c-Myc positive cells				Ki67 positive cells			
	Control		Fubp1 siRNA		Control		Fubp1 siRNA	
	IEE	OEE	IEE	OEE	IEE	OEE	IEE	OEE
**AVERAGE**	6	8	23	19	17	23	32	28
**STDEV**	0.69	1.21	1.09	0.96	0.96	1.36	1.22	1.71
***p*-value**	0.23	0.17			0.06	0.24		

Supplementary Figure S1. Statistical analysis of c-Myc and Ki67 positive cells. Statistical analysis shows increased and decreased c-Myc and Ki67 positive cell numbers along the IEE and OEE respectively in the *Fubp1* knock-down specimen. OEE, outer enamel epithelium; IEE, inner enamel epithelium.

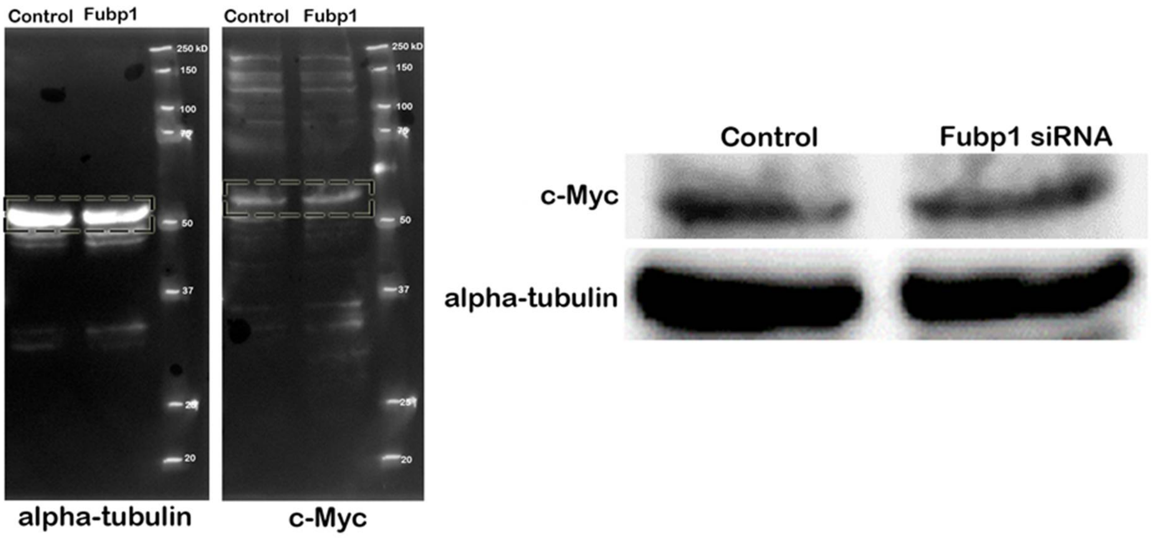

Supplementary Figure S2. Protein expression level in *Fubp1* knock-down tooth. Western blot shows no obvious changes in the protein expression level of c-Myc after *Fubp1* knock-down at E14 for 48 h. Dotted boxes demarcate the cropped regions for discussion in this study.

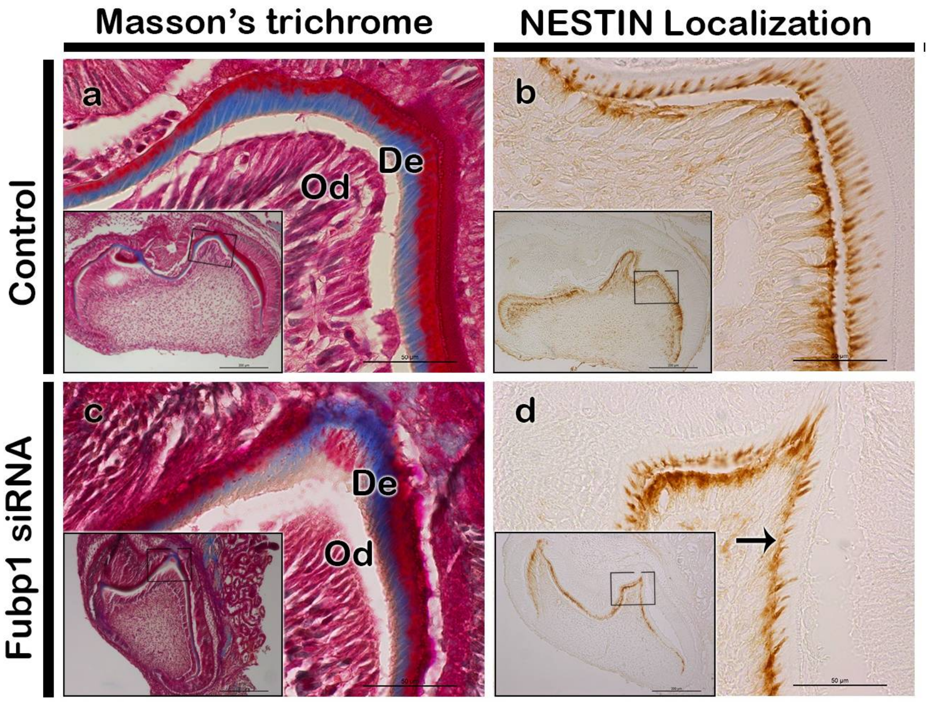

Supplementary Figure S3. One-week renal calcified tooth. Masson's trichrome staining showing one week renal calcified teeth (a, c). The localization of NESTIN is abrogated and weaker along the odontoblast layer and its processes in the *Fubp1* knock-down tooth (arrow, d) when compared with the control (b). Square box indicates an enlarged view (a-d). De; dentin, Od; odontoblast. Scale bars: 50 µm (a-d).

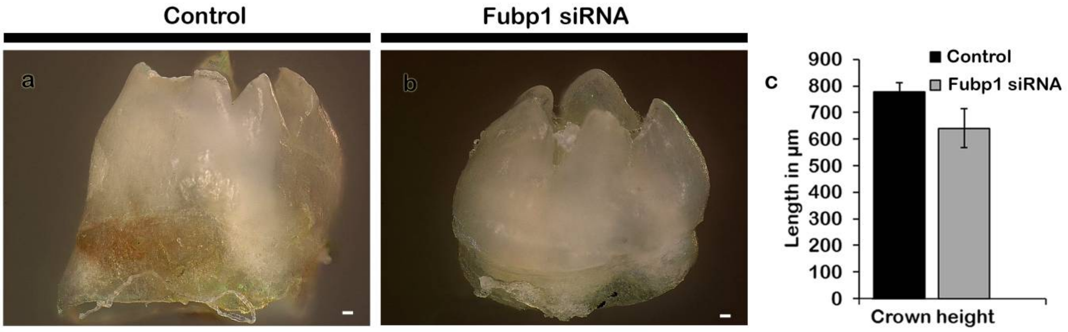

Supplementary Figure S4. Altered crown height in *Fubp1* knock-down tooth. Three-week renal calcified tooth showing decreased crown height in the *Fubp1* knock-down tooth compared with control (a-c). Scale bars 100 µm (a- b). Statistical analysis shows the decreased crown height in the *Fubp1* knock-down tooth compared with the control (N=5) (c).

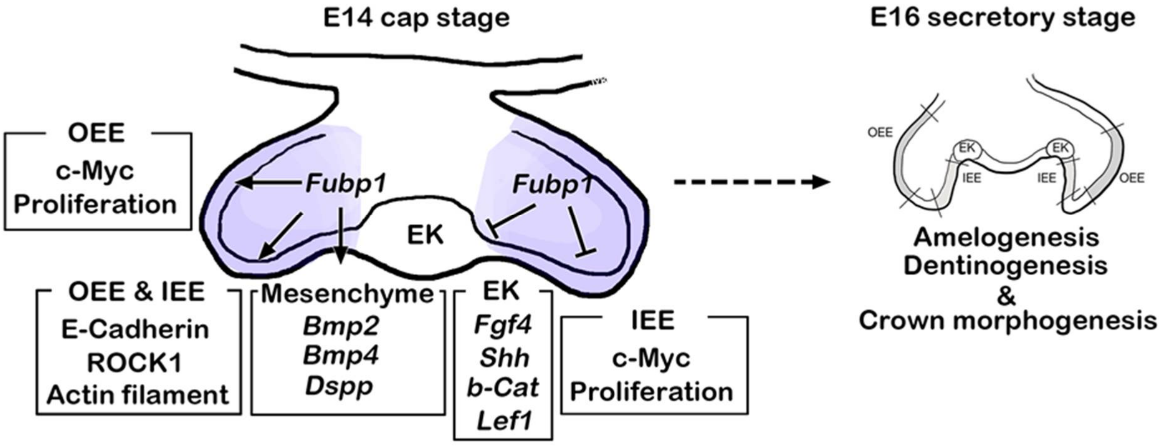

Supplementary Figure S5. Schematic diagram for developmental function of *Fubp1* at cap stage. Purple color indicates expression pattern of *Fubp1* in developing tooth germ at E14. Arrows and blunt arrows indicate the activation and inhibition respectively.

Acknowledgements

This study was supported by the National Research Foundation of Korea (grant NRF-2018R1A2A3075600, 2017R1A5A2015391 and 2015R1A2A2A01008180) funded by the Ministry of Education, Science and Technology, Republic of Korea.

Author contributions:

Yam Prasad Aryal: Conceptualization, methodology, investigation, visualization, formal analysis, writing-original draft, funding acquisition

Sanjiv Neupane: Resources, investigation, visualization, writing review and editing

Tae-Young Kim: Resources, investigation, visualization

Eui-Seon Lee: Resources, investigation, visualization

Nitin Kumar Pokhrel: Resources, investigation, visualization

Chang-Yeol Yeon: Resources, investigation, visualization

Ji-Youn Kim: Validation, resources, formal analysis, writing review and editing

Chang-Hyeon An: Validation, resources, supervision, writing review and editing

Seo-Young An: Validation, resources, writing review and editing

Eui-Kyun Park: Validation, resources, writing review and editing

Jung-Hong Ha: Validation, resources, writing review and editing

Jae-Kwang Jung: Validation, resources, writing review and editing

Hitoshi Yamamoto: Validation, resources, formal analysis, writing review and editing

Sung-Won Cho: Validation, resources, formal analysis, writing review and editing

Sanggyu Lee: Validation, resources, formal analysis, writing review and editing

Do-Yeon Kim: Validation, resources, formal analysis, writing review and editing

Tae-Yub Kwon: Validation, resources, formal analysis, writing review and editing

Youngkyun Lee: Validation, resources, supervision, writing review and editing

Wern-Joo Sohn: Validation, resources, supervision, writing review and editing

Jae-Young Kim: Conceptualization, methodology, investigation, visualization, formal analysis, writing-original draft, supervision, project administration, funding acquisition.

The authors declare no potential conflicts of interest with respect to the authorship and/or publication of this article.

References

1. Gilbert, S.F.; Barresi, M.J.F. DEVELOPMENTAL BIOLOGY, 11TH EDITION 2016. *Am. J. Med. Genet. Part A* **2017**, *173*, 1430–1430, doi:10.1002/ajmg.a.38166.

2. Kuure, S.; Vuolteenaho, R.; Vainio, S. Kidney morphogenesis: Cellular and molecular regulation. *Mech. Dev.* **2000**, *92*, 31–45, doi:10.1016/S0925-4773(99)00323-8.

3. Pispa, J.; Thesleff, I. Mechanisms of ectodermal organogenesis. *Dev. Biol.* **2003**, *262*, 195–205, doi:10.1016/S0012-1606(03)00325-7.

4. Hogan, B.L.M.; Yingling, J.M. Epithelial/mesenchymal interactions and branching morphogenesis of the lung. *Curr. Opin. Genet. Dev.* **1998**, *8*, 481–486, doi:10.1016/S0959-437X(98)80121-4.

5. Mousavi, S.J.; Hamdy Doweidar, M. Role of mechanical cues in cell differentiation and proliferation: A 3D numerical model. *PLoS One* **2015**, *10*, 1–23, doi:10.1371/journal.pone.0124529.

6. Thesleff, I. The genetic basis of tooth development and dental defects. *Am. J. Med. Genet. Part A* **2006**, *140A*, 2530–2535, doi:10.1002/ajmg.a.31360.

7. Du, W.; Hu, J.K.-H.; Du, W.; Klein, O.D. Lineage tracing of epithelial cells in developing teeth reveals two strategies for building signaling centers. *J. Biol. Chem.* **2017**, *292*, 15062–15069, doi:10.1074/jbc.M117.785923.

8. Nirvani, M.; Khuu, C.; Utheim, T.P.; Hollingen, H.S.; Amundsen, S.F.; Sand, L.P.; Sehic, A. Circadian rhythms and gene expression during mouse molar tooth development. *Acta Odontol. Scand.* **2017**, *75*, 144–153, doi:10.1080/00016357.2016.1271999.

9. Aurrekoetxea, M.; Irastorza, I.; García-Gallastegui, P.; Jiménez-Rojo, L.; Nakamura, T.; Yamada, Y.; Ibarretxe, G.; Unda, F.J. Wnt/β-Catenin Regulates the Activity of Epiprofin/Sp6, SHH, FGF, and BMP to Coordinate the Stages of Odontogenesis. *Front. cell Dev. Biol.* **2016**, *4*, 25, doi:10.3389/fcell.2016.00025.

10. Chung, H.-J.; Liu, J.; Dundr, M.; Nie, Z.; Sanford, S.; Levens, D. FBPs are calibrated molecular tools to adjust gene expression. *Mol. Cell. Biol.* **2006**, *26*, 6584–97,

doi:10.1128/MCB.00754-06.

11. He, L.; Liu, J.; Collins, I.; Sanford, S.; O'Connell, B.; Benham, C.J.; Levens, D. Loss of FBP function arrests cellular proliferation and extinguishes c-myc expression. *EMBO J.* **2000**, *19*, 1034–44, doi:10.1093/emboj/19.5.1034.

12. Zhang, J.; Chen, Q.M. Far upstream element binding protein 1: a commander of transcription, translation and beyond. *Oncogene* **2013**, *32*, 2907–2916, doi:10.1038/onc.2012.350.

13. Nie, Z.; Hu, G.; Wei, G.; Cui, K.; Yamane, A.; Resch, W.; Wang, R.; Green, D.R.; Tessarollo, L.; Casellas, R.; et al. c-Myc is a universal amplifier of expressed genes in lymphocytes and embryonic stem cells. Cell 151:68–79. NIH Public Access. **2013**, *151*, 68–79, doi:10.1016/j.cell.2012.08.033.

14. Dang, C. V. c-Myc Target Genes Involved in Cell Growth, Apoptosis, and Metabolism. *Mol. Cell. Biol.* **1999**, *19*, 1–11, doi:10.1128/mcb.19.1.1.

15. Davis-Smyth, T.; Duncan, R.C.; Zheng, T.; Michelotti, G.; Levens, D. The far upstream element-binding proteins comprise an ancient family of single-strand DNA-binding transactivators. *J. Biol. Chem.* **1996**, *271*, 31679–87, doi:10.1074/jbc.271.49.31679.

16. Chung, H.; Levens, D. Minireview Molecules and c-myc Expression : Keep the Noise Down ! **2005**, *20*, 157–166.

17. Duan, J.; Bao, X.; Ma, X.; Zhang, Y.; Ni, D.; Wang, H.; Zhang, F.; Du, Q.; Fan, Y.; Chen, J.; et al. Upregulation of far upstream element-binding protein 1 (FUBP1) promotes tumor proliferation and tumorigenesis of clear cell renal cell carcinoma. *PLoS One* **2017**, *12*, 1–16, doi:10.1371/journal.pone.0169852.

18. Hwang, I.; Cao, D.; Na, Y.; Kim, D.-Y.; Zhang, T.; Yao, J.; Oh, H.; Hu, J.; Zheng, H.; Yao, Y.; et al. Far Upstream Element-Binding Protein 1 Regulates LSD1 Alternative Splicing to Promote Terminal Differentiation of Neural Progenitors. *Stem cell reports* **2018**, *10*, 1208–1221, doi:10.1016/j.stemcr.2018.02.013.

19. Adhikari, N.; Neupane, S.; Gwon, G.-J.; Kim, J.-Y.; An, C.-H.; Lee, S.; Sohn, W.-J.; Lee,

Y.; Kim, J.-Y. Grhl3 modulates epithelial structure formation of the circumvallate papilla during mouse development. Histochem. Cell Biol. 2017, 147, 5–16, doi:10.1007/s00418-016-1487-7.

20. Neupane, S.; Sohn, W.-J.; Rijal, G.; Lee, Y.-J.; Lee, S.; Yamamoto, H.; An, C.-H.; Cho, S.-W.; Lee, Y.; Shin, H.-I.; et al. Developmental regulations of Perp in mice molar morphogenesis. Cell Tissue Res. 2014, 358, 109–121, doi:10.1007/s00441-014-1908-7.

21. Balic, A. Concise Review: Cellular and Molecular Mechanisms Regulation of Tooth Initiation. *Stem Cells* **2019**, *37*, 26–32, doi:10.1002/stem.2917.

22. Uchibe, K.; Shimizu, H.; Yokoyama, S.; Kuboki, T.; Asahara, H. Identification of novel transcription-regulating genes expressed during murine molar development. *Dev. Dyn.* **2012**, *241*, 1217–1226, doi:10.1002/dvdy.23808.

23. Zhou, W.; Chung, Y.J.; Parrilla Castellar, E.R.; Zheng, Y.; Chung, H.J.; Bandle, R.; Liu, J.; Tessarollo, L.; Batchelor, E.; Aplan, P.D.; et al. Far upstream element binding protein plays a crucial role in embryonic development, hematopoiesis, and stabilizing myc expression levels. *Am. J. Pathol.* **2016**, *186*, 701–715, doi:10.1016/j.ajpath.2015.10.028.

24. Wilson, A.; Murphy, M.J.; Oskarsson, T.; Kaloulis, K.; Bettess, M.D.; Oser, G.M.; Pasche, A.-C.; Knabenhans, C.; Macdonald, H.R.; Trumpp, A. c-Myc controls the balance between hematopoietic stem cell self-renewal and differentiation. *Genes Dev.* **2004**, *18*, 2747–63, doi:10.1101/gad.313104.

25. Luo, W.; Chen, J.; Li, L.; Ren, X.; Cheng, T.; Lu, S.; Lawal, R.A.; Nie, Q.; Zhang, X.; Hanotte, O. c-Myc inhibits myoblast differentiation and promotes myoblast proliferation and muscle fibre hypertrophy by regulating the expression of its target genes, miRNAs and lincRNAs. *Cell Death Differ.* **2019**, *26*, 426–442, doi:10.1038/s41418-018-0129-0.

26. Melnik, S.; Werth, N.; Boeuf, S.; Hahn, E.-M.; Gotterbarm, T.; Anton, M.; Richter, W. Impact of c-MYC expression on proliferation, differentiation, and risk of neoplastic transformation of human mesenchymal stromal cells. *Stem Cell Res. Ther.* **2019**, *10*, 73, doi:10.1186/s13287-019-1187-z.

27. Wen, H.; Ma, H.; Li, P.; Zheng, J.; Yu, Y.; Lv, G. Expression of far upstream element-binding protein 1 correlates with c-Myc expression in sacral chordomas and is associated with tumor progression and poor prognosis. *Biochem. Biophys. Res. Commun.* **2017**, *491*, 1047–1054, doi:10.1016/j.bbrc.2017.08.008.

28. Wang, B.; Fan, P.; Zhao, J.; Wu, H.; Jin, X.; Wu, H. FBP1 loss contributes to BET inhibitors resistance by undermining c-Myc expression in pancreatic ductal adenocarcinoma. *J. Exp. Clin. Cancer Res.* **2018**, *37*, 224, doi:10.1186/s13046-018-0888-y.

29. Rabenhorst, U.; Beinoraviciute-Kellner, R.; Brezniceanu, M.-L.; Joos, S.; Devens, F.; Lichter, P.; Rieker, R.J.; Trojan, J.; Chung, H.-J.; Levens, D.L.; et al. Overexpression of the far upstream element binding protein 1 in hepatocellular carcinoma is required for tumor growth. *Hepatology* **2009**, *50*, 1121–1129, doi:10.1002/hep.23098.

30. Ruijtenberg, S.; van den Heuvel, S. Coordinating cell proliferation and differentiation: Antagonism between cell cycle regulators and cell type-specific gene expression. *Cell Cycle* **2016**, *15*, 196–212, doi:10.1080/15384101.2015.1120925.

31. Balic, A.; Thesleff, I. Tissue Interactions Regulating Tooth Development and Renewal. In; 2015; pp. 157–186.

32. Jani, P.; Liu, C.; Zhang, H.; Younes, K.; Benson, M.D.; Qin, C. The role of bone morphogenetic proteins 2 and 4 in mouse dentinogenesis. *Arch. Oral Biol.* **2018**, *90*, 33–39, doi:10.1016/j.archoralbio.2018.02.004.

33. Gluhak-Heinrich, J.; Guo, D.; Yang, W.; Harris, M.A.; Lichtler, A.; Kream, B.; Zhang, J.; Feng, J.Q.; Smith, L.C.; Dechow, P.; et al. New roles and mechanism of action of BMP4 in postnatal tooth cytodifferentiation. *Bone* **2010**, *46*, 1533–1545, doi:10.1016/j.bone.2010.02.024.

34. Feng, J.; Yang, G.; Yuan, G.; Gluhak-Heinrich, J.; Yang, W.; Wang, L.; Chen, Z.; Schulze McDaniel, J.; Donly, K.J.; Harris, S.E.; et al. Abnormalities in the Enamel in Bmp2-Deficient Mice. *Cells Tissues Organs* **2011**, *194*, 216–221, doi:10.1159/000324644.

35. Li, L.; Tang, Q.; Kwon, H.-J.E.; Wu, Z.; Kim, E.-J.; Jung, H.-S. An Explanation for How

FGFs Predict Species-Specific Tooth Cusp Patterns. *J. Dent. Res.* **2018**, *97*, 828–834, doi:10.1177/0022034518759625.

36. Hamidi, K.; Darvish, J.; Matin, M.M.; Javanmard, A.S.; Kilpatrick, C.W. Tooth Morphogenesis and FGF4 Expression During Development of Molar Tooth in Three Muroid Rodents: Calomyscus elburzensis (Calomyscidae), Mesocricetus auratus (Cricetidae) and Mus musculus (Muridae). *Anat. Rec.* **2017**, *300*, 2138–2149, doi:10.1002/ar.23678.

37. Cho, S.-W.; Lee, H.-A.; Cai, J.; Lee, M.-J.; Kim, J.-Y.; Ohshima, H.; Jung, H.-S. The primary enamel knot determines the position of the first buccal cusp in developing mice molars. *Differentiation.* **2007**, *75*, 441–51, doi:10.1111/j.1432-0436.2006.00153.x.

38. Shi, C.; Yuan, Y.; Guo, Y.; Jing, J.; Ho, T.V.; Han, X.; Li, J.; Feng, J.; Chai, Y. BMP Signaling in Regulating Mesenchymal Stem Cells in Incisor Homeostasis. *J. Dent. Res.* **2019**, *98*, 904–911, doi:10.1177/0022034519850812.

39. Zhang, L.; Hua, F.; Yuan, G.-H.; Zhang, Y.-D.; Chen, Z. Sonic hedgehog signaling is critical for cytodifferentiation and cusp formation in developing mouse molars. *J. Mol. Histol.* **2008**, *39*, 87–94, doi:10.1007/s10735-007-9132-3.

40. Otsu, K.; Harada, H. Rho GTPases in ameloblast differentiation. *Jpn. Dent. Sci. Rev.* **2016**, *52*, 32–40, doi:10.1016/j.jdsr.2015.09.001.

41. Huang, X.; Xu, X.; Bringas, P.; Hung, Y.P.; Chai, Y. Smad4-Shh-Nfic signaling cascade-mediated epithelial-mesenchymal interaction is crucial in regulating tooth root development. *J. Bone Miner. Res.* **2010**, *25*, 1167–1178, doi:10.1359/jbmr.091103.

42. van Dam, S.; Võsa, U.; van der Graaf, A.; Franke, L.; de Magalhães, J.P. Gene co-expression analysis for functional classification and gene-disease predictions. *Brief. Bioinform.* **2018**, *19*, 575–592, doi:10.1093/bib/bbw139.

43. Hatakeyama, J.; Fukumoto, S.; Nakamura, T.; Haruyama, N.; Suzuki, S.; Hatakeyama, Y.; Shum, L.; Gibson, C.W.; Yamada, Y.; Kulkarni, A.B. Synergistic roles of amelogenin and ameloblastin. *J. Dent. Res.* **2009**, *88*, 318–22, doi:10.1177/0022034509334749.

44. Verdelis, K.; Szabo-Rogers, H.L.; Xu, Y.; Chong, R.; Kang, R.; Cusack, B.J.; Jani, P.; Boskey, A.L.; Qin, C.; Beniash, E. Accelerated enamel mineralization in Dspp mutant mice. *Matrix Biol.* **2016**, *52–54*, 246–259, doi:10.1016/j.matbio.2016.01.003.

45. Zhang, H.; Xie, X.; Liu, P.; Liang, T.; Lu, Y.; Qin, C. Transgenic expression of dentin phosphoprotein (DPP) partially rescued the dentin defects of DSPP-null mice. *PLoS One* **2018**, *13*, e0195854, doi:10.1371/journal.pone.0195854.

46. Walker, J.L.; Menko, A.S.; Khalil, S.; Rebustini, I.; Hoffman, M.P.; Kreidberg, J.A.; Kukuruzinska, M.A. Diverse roles of E-cadherin in the morphogenesis of the submandibular gland: Insights into the formation of acinar and ductal structures. *Dev. Dyn.* **2008**, *237*, 3128–3141, doi:10.1002/dvdy.21717.

47. Park, S.Y.; Shin, J.-H.; Kee, S.-H. E-cadherin expression increases cell proliferation by regulating energy metabolism through nuclear factor-κB in AGS cells. *Cancer Sci.* **2017**, *108*, 1769–1777, doi:10.1111/cas.13321.

48. Kilkenny, C.; Browne, W.J.; Cuthill, I.C.; Emerson, M.; Altman, D.G. Improving Bioscience Research Reporting: The ARRIVE Guidelines for Reporting Animal Research. *PLoS Biol.* **2010**, *8*, e1000412, doi:10.1371/journal.pbio.1000412.

49. Cai, J.; Cho, S.-W.; Kim, J.-Y.; Lee, M.-J.; Cha, Y.-G.; Jung, H.-S. Patterning the size and number of tooth and its cusps. *Dev. Biol.* **2007**, *304*, 499–507, doi:10.1016/j.ydbio.2007.01.002.

50. Jung, J.K.; Gwon, G.J.; Neupane, S.; Sohn, W.J.; Kim, K.R.; Kim, J.Y.; An, S.Y.; Kwon, T.Y.; An, C.H.; Lee, Y.; et al. Bortezomib Facilitates Reparative Dentin Formation after Pulp Access Cavity Preparation in Mouse Molar. *J. Endod.* **2017**, *43*, 2041–2047, doi:10.1016/j.joen.2017.07.018.
